# Supplementary material for: Systematic Review of the Short-Term versus Long-Term Duration of Antibiotic Management for Neutropenic Fever in Patients with Cancer
Source: Cancers (Basel). 2023 Mar 5;15(5):1611. doi: 10.3390/cancers15051611 (PMC10001032; doi:10.3390/cancers15051611)
Supplement: Supplementary file 1 [file cancers-15-01611-s001.zip › cancers-2181732-supplementary.pdf]

Keywords to search the case report of short-term duration versus long-term in febrile neutropenia

A literature search was conducted

- CENTRAL(September 30, 2022)

#1 MeSH descriptor: [Neutropenia] explode all trees

#2 neutropaeni* or neutropeni* or granulocytopeni* or granulocytopaeni*

#3 #1 or #2

#4 MeSH descriptor: [Anti-Infective Agents] explode all trees

#5 antibiotic* or antimicrobial* or anti-microbial* or antibacterial* or anti-bacterial*

#6 #4 or #5

#7 MeSH descriptor: [] explode all trees and with qualifier(s): [administration & dosage - AD]

#8 duration or continue* or continuation or carry on or discontinue* or discontinuation or stop*

#9 #7 or #8

#10 #3 and #7 and #9

#11 #10 with Cochrane Library publication date from Jan 2018 to present, in Trials

- Embase (September 30, 2022)

(('neutropenia'/exp OR (neutropaeni* OR neutropeni* OR granulocytopeni* OR granulocytopaeni*))

AND ('antiinfective agent'/exp OR (antibiotic* OR antimicrobial* OR anti-microbial* OR

antibacterial* OR anti-bacterial*)) AND ((duration OR continue* OR continuation OR carry) AND on OR discontinue* OR discontinuation OR stop*)) AND ('crossover procedure'/exp OR 'double blind procedure'/exp OR 'randomized controlled trial'/exp OR 'single blind procedure'/exp OR random* OR factorial* OR ((crossover* OR cross) AND over* OR cross-over*) OR placebo* OR double* NEXT/1 blind* OR singl* NEXT/1 blind* OR assign* OR allocat* OR volunteer*) AND [2018-2022]/py

- Medline (September 30, 2022)

1. Neutropenia/

2. (neutropaeni* or neutropeni* or granulocytopeni* or granulocytopaeni*).mp.

3. 1 or 2

4. exp Anti-Infective Agents/

5. (antibiotic* or antimicrobial* or anti-microbial* or antibacterial* or anti-bacterial*).mp.

6. 4 or 5

7. (duration or continue* or continuation or carry on or discontinue* or discontinuation or stop*).mp.

8. 3 and 6 and 7

9. randomized controlled trial.pt.

10. controlled clinical trial.pt.

11. randomized.ab.

12. placebo.ab.

13. clinical trials as topic.sh.

14. randomly.ab.

15. trial.ti.

16. 9 or 10 or 11 or 12 or 13 or 14 or 15

17. 8 and 16

18. (animals not (humans and animals)).sh.

19. 17 not 18

Study or Subgroup	short antibiotic therapy Events	Total	long antibiotic therapy Events	Total	Weight	Risk Ratio M-H, Fixed, 95% CI
Aguilar-Guisado 2017	1	78	3	79	41.3%	0.34 [0.04, 3.18]
Jonge 2022	5	144	1	137	14.2%	4.76 [0.56, 40.20]
Ram 2021	2	59	3	51	44.5%	0.58 [0.10, 3.31]
Santolaya 2017	0	84	0	92		Not estimable
**Total (95% CI)**		**365**		**359**	**100.0%**	**1.07 [0.39, 2.92]**
Total events	8		7			

Heterogeneity: Chi² = 3.38, df = 2 (P = 0.18); I² = 41%
Test for overall effect: Z = 0.13 (P = 0.89)

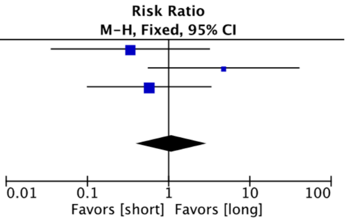

Study or Subgroup	short antibiotic therapy Events	Total	long antibiotic therapy Events	Total	Weight	Risk Ratio M-H, Fixed, 95% CI
Aguilar-Guisado 2017	11	78	14	79	27.2%	0.80 [0.39, 1.64]
Jonge 2022	28	144	21	137	42.1%	1.27 [0.76, 2.12]
Ram 2021	9	59	12	51	25.2%	0.65 [0.30, 1.41]
Santolaya 2017	4	84	3	92	5.6%	1.46 [0.34, 6.33]
**Total (95% CI)**		**365**		**359**	**100.0%**	**0.99 [0.70, 1.42]**
Total events	52		50			

Heterogeneity: Chi² = 2.64, df = 3 (P = 0.45); I² = 0%
Test for overall effect: Z = 0.03 (P = 0.98)

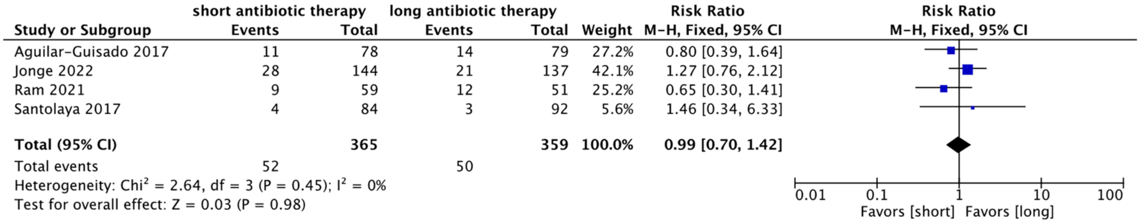

Study or Subgroup	short antibiotic therapy Events	Total	long antibiotic therapy Events	Total	Weight	Risk Ratio M-H, Fixed, 95% CI
Aguilar-Guisado 2017	9	78	12	79	83.3%	0.76 [0.34, 1.70]
Jonge 2022	15	114	13	98	0.0%	0.99 [0.45, 2.20]
Santolaya 2017	0	84	2	92	16.7%	0.22 [0.01, 4.49]
**Total (95% CI)**		**162**		**171**	**100.0%**	**0.67 [0.31, 1.45]**
Total events	9		14			

Heterogeneity: Chi² = 0.62, df = 1 (P = 0.43); I² = 0%
Test for overall effect: Z = 1.02 (P = 0.31)

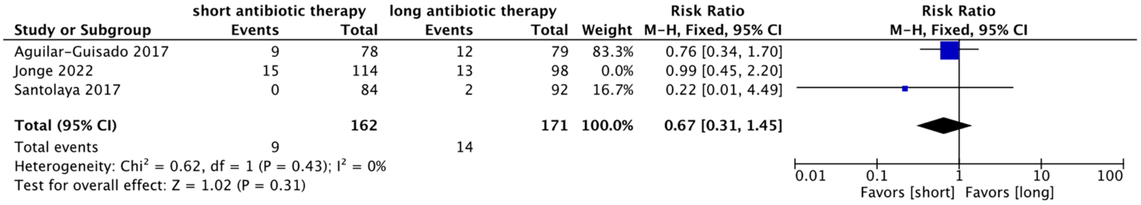

Supplemental Figure S1. Summary of findings of short-term duration of antibiotics compared with long-term duration of antibiotics presenting Forest plot for only hematological malignancy patients including stem cell transplantation- Jonge et al. [4], Ram et al. [14], Aguilar-Guisado 2017 [8], and Santolaya et al.[16], mortality(above), clinical failure(middle), bacteremia(below). Total risk ration across 1 (left favors the short, right favors long)
abbreviation: RCT; randomized control study, CI; confidence interval.

Study or Subgroup	short antibiotic therapy Events	Total	long antibiotic therapy Events	Total	Weight	Risk Ratio M-H, Fixed, 95% CI
Aguilar-Guisado 2017	1	78	3	79	41.3%	0.34 [0.04, 3.18]
Jonge 2022	5	144	1	137	14.2%	4.76 [0.56, 40.20]
Klaassen 2000	0	36	0	37		Not estimable
Kumar 2020	0	38	0	37		Not estimable
Ram 2021	2	59	3	51	44.5%	0.58 [0.10, 3.31]
**Total (95% CI)**		**355**		**341**	**100.0%**	**1.07 [0.39, 2.92]**
Total events	8		7			

Heterogeneity: Chi² = 3.38, df = 2 (P = 0.18); I² = 41%
Test for overall effect: Z = 0.13 (P = 0.89)

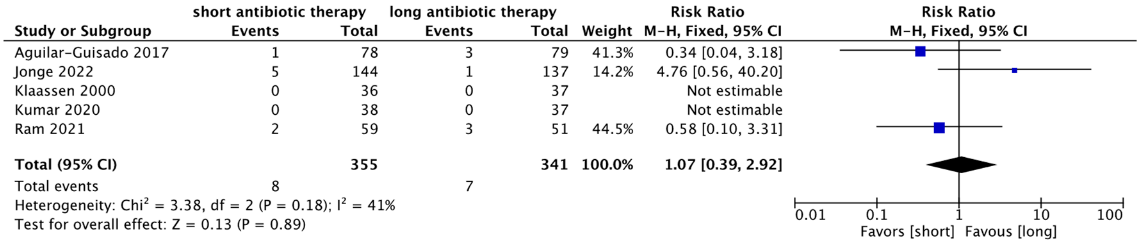

Study or Subgroup	short antibiotic therapy Events	Total	long antibiotic therapy Events	Total	Weight	Risk Ratio M-H, Fixed, 95% CI
Bjornsson 1977	3	6	2	11	15.3%	2.75 [0.62, 12.17]
Pizzo 1979	2	17	0	16	5.6%	4.72 [0.24, 91.41]
Pizzo 1982	5	16	8	34	55.5%	1.33 [0.52, 3.42]
Rodriguez 1973	3	12	2	10	23.6%	1.25 [0.26, 6.07]
Santolaya 1997	0	36	0	39		Not estimable
Santolaya 2017	0	84	0	92		Not estimable
**Total (95% CI)**		**171**		**202**	**100.0%**	**1.72 [0.86, 3.41]**
Total events	13		12			

Heterogeneity: Chi² = 1.27, df = 3 (P = 0.74); I² = 0%
Test for overall effect: Z = 1.54 (P = 0.12)

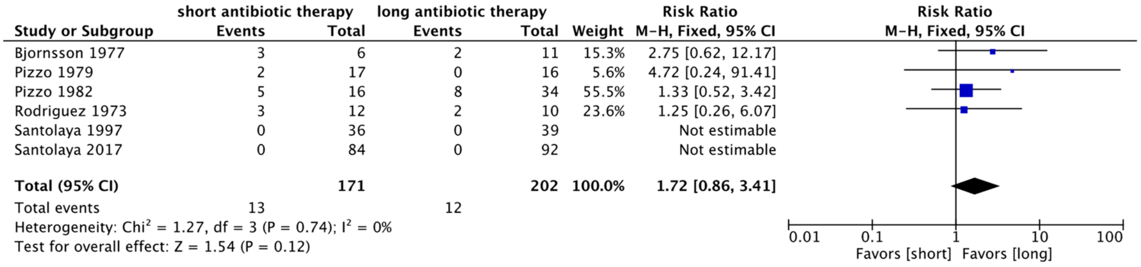

Supplemental Figure S2. Summary of findings of short-term duration of antibiotics compared with long-term duration of antibiotics presenting Forest plot for mortality in low(above) and unclear(below) allocation concealment bias as sensitivity analysis..
abbreviation: RCT; randomized control study, CI; confidence interval.
